# Phosphodiesterase 5 inhibitor mirodenafil ameliorates Alzheimer-like pathology and symptoms by multimodal actions

**DOI:** 10.1186/s13195-022-01034-3

**Published:** 2022-07-08

**Authors:** Byung Woo Kang, Fred Kim, Joon-Yong Cho, SangYun Kim, Jinseol Rhee, Jai Jun Choung

**Affiliations:** 1 Pharmacology Team, AriBio Co., Ltd, 56 Dongpangyo-ro, Bundang-gu, Seongnam-si, Gyeonggi-do 13535 Republic of Korea; 2 Department of Exercise Biochemistry, Korea National Sport University, Seoul, 05541 Republic of Korea; 3 Department of Neurology, Seoul National University College of Medicine & Seoul National University Bundang Hospital, 82 Gumi-ro 173-beongil, Bundang-gu, Seongnam-si, Gyeonggi-do 13620 Republic of Korea

**Keywords:** Alzheimer’s disease, Phosphodiesterase 5, Mirodenafil, Amyloid-β, tau, Apoptosis, Autophagy, Glucocorticoid receptor, Dickkopf-1, Wnt/β-catenin

## Abstract

**Background:**

Alzheimer’s disease (AD) pathology is associated with complex interactions among multiple factors, involving an intertwined network of various signaling pathways. The polypharmacological approach is an emerging therapeutic strategy that has been proposed to overcome the multifactorial nature of AD by targeting multiple pathophysiological factors including amyloid-β (Aβ) and phosphorylated tau. We evaluated a blood-brain barrier penetrating phosphodiesterase 5 (PDE5) inhibitor, mirodenafil (5-ethyl-2-7-n-propyl-3,5-dihydrro-4H-pyrrolo[3,2-d]pyrimidin-4-one), for its therapeutic effects on AD with polypharmacological properties.

**Methods:**

To evaluate the potential of mirodenafil as a disease-modifying AD agent, mirodenafil was administered to test its effects on the cognitive behaviors of the APP-C105 AD mouse model using the Morris water maze and passive avoidance tests. To investigate the mechanisms of action that underlie the beneficial disease-modifying effects of mirodenafil, human neuroblastoma SH-SY5Y cells and mouse hippocampal HT-22 cells were used to show mirodenafil-induced alterations associated with the cyclic guanosine monophosphate (cGMP)/cGMP-dependent protein kinase (PKG)/cAMP-responsive element-binding protein (CREB) pathway, apoptotic cell death, tau phosphorylation, amyloidogenesis, the autophagy-lysosome pathway, glucocorticoid receptor (GR) transcriptional activity, and the Wnt/β-catenin signaling.

**Results:**

Here, mirodenafil is demonstrated to improve cognitive behavior in the APP-C105 mouse model. Mirodenafil not only reduced the Aβ and phosphorylated tau burdens in vivo, but also ameliorated AD pathology induced by Aβ through the modulation of the cGMP/PKG/CREB signaling pathway, glycogen synthase kinase 3β (GSK-3β) activity, GR transcriptional activity, and the Wnt/β-catenin signaling in neuronal cells. Interestingly, homodimerization and nuclear localization of GR were inhibited by mirodenafil, but not by other PDE5 inhibitors. In addition, only mirodenafil reduced the expression levels of the Wnt antagonist Dickkopf-1 (Dkk-1), thus activating the Wnt/β-catenin signaling.

**Conclusions:**

These findings strongly suggest that the PDE5 inhibitor mirodenafil shows promise as a potential polypharmacological drug candidate for AD treatment, acting on multiple key signaling pathways involved in amyloid deposition, phosphorylated tau burden, the cGMP/PKG/CREB pathway, GSK-3β kinase activity, GR signaling, and the Wnt/β-catenin signaling. Mirodenafil administration to the APP-C105 AD mouse model also improved cognitive behavior, demonstrating the potential of mirodenafil as a polypharmacological AD therapeutic agent.

**Supplementary Information:**

The online version contains supplementary material available at 10.1186/s13195-022-01034-3.

## Background

Alzheimer’s disease (AD) is a devastating neurodegenerative disorder, most often associated with memory deficits and cognitive dysfunction, resulting in an enormous socio-economic burden on caregivers and the public health sector worldwide [1]. There is an urgent need to develop disease-modifying therapies for AD that may slow the rate of disease progression. Drug development for AD has been one of the most challenging therapeutic areas with unsustainable costs and frequent failures in clinical trials [2]. The high failure rates in late-stage clinical trials raised doubts about the classical paradigm “one-drug, one-target, one-disease” for AD drug development. Instead, recent advances in network pharmacology held the promise of employing a polypharmacological approach for multi-target drug discovery [3].

AD is characterized by the cerebral aggregation of amyloid-β (Aβ) and phosphorylated tau, which accumulate and form amyloid plaques and neurofibrillary tangles, respectively [4]. In addition, the complex pathogenesis of AD includes impaired neurotransmission, increased oxidative stress, reduced cerebral blood flow (CBF), or neuroinflammation, which may be partially responsible for high failure rates in AD clinical trials. Thus, polypharmacological approaches directed against AD-related multiple molecular pathways may be more promising for the development of new therapeutic agents for AD.

Aberrant accumulation of Aβ or tau exerts multiple effects on molecular pathways at different stages of AD progression. For example, Aβ oligomers interfere with normal neurotransmission by altering glutamate receptor-dependent signaling cascades at the nerve synapses, thereby leading to long-term potentiation (LTP) impairment [5]. Aβ markedly impairs hippocampal LTP by downregulating the nitric oxide (NO)/soluble guanylyl cyclase (sGC)/cGMP/cAMP-responsive element-binding protein (CREB) cascade, resulting in CREB phosphorylation [6]. In addition, Aβ-dependent and Aβ-independent vascular dysfunction contributes to AD pathogenesis [7].

Phosphodiesterase 5 (PDE5) mediates the hydrolysis of the cyclic nucleotide cGMP into its inactive form. Notably, levels of PDE5 in the temporal cortex of AD patients are significantly higher than those of age-matched healthy control subjects [8]. Inhibition of PDE5 suppresses cGMP hydrolysis, resulting in the activation of protein kinase G (PKG) and increased phosphorylation of CREB at Ser133, which rescues LTP impairment and cognitive dysfunction in an amyloid precursor protein/presenilin-1 (APP/PS1) AD mouse model [9]. Furthermore, PDE5 inhibitors improve spatial learning and memory in several AD mouse models, suggesting that PDE5 is a viable therapeutic target for AD [10].

The PDE5 inhibitor sildenafil improves memory function and reverses cognitive deficits by restoring CREB signaling in APP/PS1 and aged Tg2576 transgenic AD mice [11, 12]. Another PDE5 inhibitor, tadalafil, enhances spatial memory by decreasing tau protein levels in J20 transgenic AD mice [10]. Moreover, the oral administration of sildenafil increases CBF and cerebral oxygen metabolism in AD patients, suggesting that PDE5 inhibitors may serve as potential therapeutic agents for AD associated with vascular dysfunction [13]. Recently, a computational analysis for identifying the relationships between drug targets and the molecular networks connecting AD-associated genes, revealed that the people who took sildenafil were 69% less likely to develop AD over 6 years. This study indicates a strong correlation between sildenafil use and reduced AD risk [14].

Repositioning of mirodenafil, a clinically approved PDE5 inhibitor with good blood-brain barrier permeability, allows us to explore new avenues for an adequate polypharmacological AD therapeutic agent [15, 16]. Based on the observed therapeutic effects associated with the use of PDE5 inhibitors against AD pathology, we hypothesized that the PDE5 inhibitor mirodenafil could be repurposed as a polypharmacological agent that directly and indirectly ameliorates pathological changes in AD. In the present study, we investigated the mechanisms of action that underlie the beneficial disease-modifying effects of mirodenafil against Aβ-induced pathology using both in vitro and in vivo AD mouse models.

## Methods

### Behavioral studies using APP-C105 transgenic mice

The APP-C105 transgenic mice, available from the Department of Laboratory Animal Resources at the National Institute of Toxicological Research, Korea Food and Drug Administration (KFDA), overexpress the c-terminal 105 amino acid fragment (C105) of amyloid precursor protein (APP) under the control of neuron-specific enolase (NSE) promoter [17]. The APP-C105 fragment mimics the carboxy-terminal fragment (β--APP-CTF) after first cleavage of APP by β-secretase in the amyloidogenic pathway, generating neurotoxic Aβ species [18]. Intracerebroventricular injection of APP-C105 fragment significantly decreased levels of acetylcholine (ACh) in the hippocampus and cerebral cortex with concurrent memory impairments, suggesting that cholinergic dysfunction by APP-C105 fragment may contribute to progressive loss of memory function [19]. Inhibition of PDE5 enhanced stimulation-induced release of synaptic ACh in the rat hippocampus [20]. Thus, the APP-C105 mouse is one of several genetic AD mouse models with cholinergic dysfunction used to test the therapeutic effects of PDE5 inhibitor for the treatment of Aβ-related memory impairments.

Mirodenafil (4 mg/kg) in PBS was administered daily via intraperitoneal (i.p.) injection to 13-month-old male APP-C105 transgenic mice (*n* = 6) for 4 weeks. PBS vehicle solution was administered to age-matched male wild-type (*n* = 6) and transgenic (*n* = 6) mice. To test hippocampus-dependent learning and memory functions, the Morris water maze experiment was conducted. Mirodenafil was daily administered by i.p. injection (4 mg/kg) after the basic acquisition training. Latency to target, distance to target, and swimming pattern were analyzed by SMART 3.0 software (Panlab) after the daily administration of mirodenafil for 4 weeks. Prior to the Morris water maze trials, each animal was subjected to pre-training, performing two consecutive trials each day for 5 days. In each trial, the animals were allowed to search for the target platform for a maximum of 60 seconds. The time required to reach the target was recorded, and the second trial was repeated after an interval of at least 5 min. To evaluate fear-conditioned learning and memory, APP-C105 mice were subjected to the passive avoidance test, and mirodenafil was administered by i.p. injection (4 mg/kg) for 4 weeks before the training trial. To monitor the safety of mirodenafil and examine effects on the central nervous system, mirodenafil was administered to a total 40 male healthy control mice aged 8 weeks, allocated to mirodenafil groups or vehicle control group, at single doses of 25, 75, or 225 mg/kg. Compared with the vehicle control group, the mirodenafil groups showed no significant differences in general physiology or behavior, including locomotor activity and the pain response (Additional File 1: Table S1). All experimental procedures were approved by the Committee on Animal Research at Korea National Sport University. All animal work and experimental procedures presented here were conducted in compliance with relevant regulations and guidelines of the ARRIVE (Animal Research: Reporting of In Vivo Experiments).

#### Cell culture and treatments

SH-SY5Y human neuroblastoma cells were obtained from the Korean Cell Line Bank (KCLB) at the Cancer Research Institute of the Seoul National University College of Medicine and cultured in Dulbecco’s modified Eagle medium: nutrient mixture F-12 (DMEM/F-12, Hyclone) containing 10% fetal bovine serum (FBS) and 1% penicillin/streptomycin (P-S) in a humidified atmosphere at 37°C and 5% CO_2_. To differentiate SH-SY5Y cells, the cell culture media was replaced with differentiation media (DMEM/F-12, 1% FBS, 10 μM retinoic acid) after 3 and 6 days of culture and prior to treatment administration. Differentiated SH-SY5Y cells were treated with oligomeric human beta-amyloid peptide (1–42, Abcam) for 72 h and then treated with mirodenafil for an additional 24 h. Subsequently, the SH-SY5Y cells were treated with either the phosphatidylinositol-3 (PI-3) kinase inhibitor LY-294002 (Sigma, #L9908) or the autophagy inhibitor 3-MA (Sigma, #M9281), and mirodenafil for an additional 24 h. For the okadaic acid treatment, differentiated SH-SY5Y cells were co-treated with mirodenafil and okadaic acid (Sigma, #O9381) for 3 h. The mature cholinergic neuron-like phenotype of differentiated SH-SY5Y cells provides an in vitro model that mimics the pathophysiology of the cholinergic neurons affected by AD [21].

HT-22 mouse hippocampal neuronal cells were purchased from Sigma-Aldrich and maintained in high-glucose DMEM (Hyclone) supplemented with 10% FBS and 1% P-S in a humidified atmosphere at 37°C and 5% CO_2_. These cells were differentiated in Neurobasal Plus medium (Gibco) containing 1× N2 supplement (Gibco) and P-S for 24 h before treatment. Differentiated HT-22 cells were co-treated with oligomeric rat/mouse beta-amyloid peptide (1–42; Abcam) and mirodenafil for 6 h. The Aβ-induced changes in the hippocampal signaling pathway were examined in differentiated HT-22 cells, which possess cholinergic neuronal properties [22].

HEK293 cells were acquired from the KCLB and cultured in low-glucose DMEM (Hyclone) containing 10% FBS and 1% P-S. HEK293 cells were co-treated with dexamethasone, mirodenafil, and CORT-108297 (Advanced ChemBlocks) for 24 h after transfection with pBIND-GR reporter gene constructs (Promega). HEK293 cells were chosen for the GR reporter assay and the GR co-immunoprecipitation experiment because they express relatively low levels of endogenous GR protein and respond to dexamethasone-induced GR transcriptional activation [23].

Primary human brain pericytes (ACBRI 498), isolated by the enzymatic dissociation of normal human cerebral cortex tissue, were purchased from Cell Systems. The pericytes were grown and passaged in Cell Systems Complete Classic Medium (4Z0-500).

#### Mirodenafil and Aβ oligomers

Mirodenafil was chemically synthesized at the Korea Research Institute of Chemical Technology [24]. Lyophilized human beta-amyloid peptide (1–42, Abcam, #ab120301) was dissolved in DMSO to make a stock solution (1 mM), divided into aliquots, and stored at −80°C. For the treatment of SH-SY5Y cells, the human Aβ_42_ aliquots were equilibrated at room temperature and diluted with DMEM/F-12 medium containing 1% FBS and 1% P-S to a final peptide concentration of 1 μM (10 μM was used only for the apoptosis experiment). To prepare oligomerized human Aβ_42_, Aβ_42_ peptide diluted with DMEM/F-12 medium was incubated at 37°C in a CO_2_ incubator for 3 h. To prepare a stock solution (200 μM) of mouse Aβ_42_ peptide, lyophilized rat/mouse beta-amyloid peptide (Abcam, #ab120959) was solubilized in distilled water, divided into aliquots, and stored at −80°C. Before the treatment of HT-22 cells, 200 μM Aβ_42_ aliquots diluted with PBS buffer were equilibrated at room temperature for 1 h to generate 100 μM mouse Aβ_42_ oligomers.

#### Quantitative measurement of cGMP

The cGMP levels were measured using a cGMP Complete ELISA Kit (Abcam), according to the manufacturer’s instructions. Briefly, SH-SY5Y cells were washed with PBS and incubated with 0.1 N HCl at room temperature for 10 min. The resulting cell lysates were centrifuged at 600 × *g* at room temperature to obtain a pellet containing cellular debris. cGMP Complete alkaline phosphatase conjugate and GMP Complete antibody were mixed with the supernatant in the wells of the cGMP Complete ELISA Kit. After incubation at room temperature for 2 h with gentle shaking, the wells were washed with 1× wash buffer. Absorbance at 405 nm was measured to determine the cGMP concentrations of the supernatant after incubation with the pNpp substrate solution at room temperature for 1 h.

#### Western blot analysis

SH-SY5Y or HT-22 cells were washed with PBS buffer and incubated on ice for 15 min with EzRIPA buffer (Atto), containing protease inhibitors and phosphatase inhibitors. Whole-cell lysates were homogenized by sonication for 15 min using Bioruptor (CosmoBio) and then centrifuged at 15,000×*g* at 4°C for 10 min. The cleared whole-cell lysates were fractionated by SDS-PAGE, transferred to polyvinylidene difluoride (PVDF) membranes, and immunoblotted using the appropriate primary antibodies and horseradish peroxidase (HRP)-conjugated secondary antibodies. Hippocampal tissue lysates from APP-C105 mice were prepared using a Dounce homogenizer in Protein Extraction Solution-RIPA (ELPIS biotech, Korea), containing protease inhibitor cocktail (Roche), and centrifuged at 14,000×*g* for 10 min at 4°C. Cleared tissue lysates were fractionated by SDS-PAGE, transferred to PVDF membranes, and blotted with the appropriate primary antibodies and HRP-conjugated secondary antibodies. The primary antibodies used in all experiments are listed in Table S1. SuperSignal™ West Femto Maximum Sensitivity Substrate (Thermo Fischer) was used as an enhanced chemiluminescent (ECL) substrate to detect protein levels. Chemiluminescent images were captured using the LuminoGraph I Imaging System (Atto). Expression levels of proteins were quantified by gel densitometry using ImageJ software (NIH).

#### Mitochondrial membrane potential assay

SH-SY5Y cells were washed with PBS buffer and incubated for 15 min at 37°C in a CO_2_ incubator with JC-1 reagent from the Mitochondrial Membrane Potential Assay Kit (Abcam). The fluorescence excitation and emission spectra were measured for both the JC-1 monomer and JC-1 aggregate using the Varioskan LUX multimode microplate reader (Thermo Fisher Scientific). The ratio of JC-1 aggregates to JC-1 monomers was used as an indicator of the mitochondrial membrane potential.

#### Real-time assessment of in vitro cytotoxicity

Cell membrane integrity was measured kinetically to detect cell death in real-time using IncuCyte® Cytotox Red Reagent (Essen Bioscience). Briefly, SH-SY5Y cells were seeded into a 96-well plate and treated with Aβ_42_ oligomers for 72 h and mirodenafil for 24 h at 37°C in a CO_2_ incubator. IncuCyte® Cytotox Red Reagent was added to each well, and cell images were captured every 90 min at 200× magnification for 24 h using the IncuCyte® S3 Live-Cell Analysis System (Satorius).

#### Real-time qRT-PCR

Total RNA was isolated from SH-SY5Y or HT-22 cells using TRIzol reagent (Molecular Research Center, Inc.), reverse-transcribed using the ImProm-II™ Reverse Transcription System (Promega), and the resulting cDNA was used for qRT-PCR. PCR primers and conditions are summarized in Table S2. qRT-PCR was performed in a QuantStudio 5 Real-Time PCR System (ThermoFisher Scientific) using SYBR Premix Ex Taq (Tli RNaseH Plus, RR420) from TaKaRa Bio Inc. All qRT-PCR reactions were conducted in duplicate, and amplification signals from individual target genes were normalized to that of glyceraldehyde-3-phosphate dehydrogenase (GAPDH).

#### Transcription factor activation profiling plate array

Differentiated HT-22 cells were treated with 1 μM Aβ_42_ oligomers and 5 μM mirodenafil for 6 h at 37°C in a CO_2_ incubator. Nuclear extracts were isolated from cells using a nuclear extraction kit (Signosis). The activation of 96 TFs was simultaneously monitored using the TF activation profiling plate array II (Signosis) according to the manufacturer’s instructions.

#### Reporter gene assays

For the GR reporter gene assay, HT-22 or HEK293 cells were seeded on a 96-well plate and co-transfected with pGL4.35[luc2P/9XGAL4UAS/Hygro] vector (Promega) and pBIND-GR (Promega) vector using Fugene HD reagent for 24 h at 37°C in a CO_2_ incubator. The transfected HT-22 cells were co-treated with 1μM Aβ_42_ oligomers and mirodenafil for an additional 6 h. The transfected HEK293 cells were co-treated with 10 μM dexamethasone, mirodenafil, and CORT-108297 for an additional 24 h. The Dual-Glo Luciferase assay system (Promega) was used to monitor luciferase activities. β-Catenin-Tcf/Lef transcriptional activity was determined using TOPFLASH. Luciferase activity was measured relative to β-galactosidase activity to normalize for transfection efficiency.

### In vitro GR binding assay

PolarScreen^TM^ GR Competitor Assay (Thermo Fischer Scientific) for a non-radioactive screening of potential GR ligands was conducted to determine the IC_50_ values of test compound (dexamethasone or mirodenafil), according to the manufacturer’s instructions. Shortly, purified GR full-length recombinant proteins were mixed with a selective fluorescent GR ligand (Fluormone GS Red) to form a GR/Fluormone GR Red complex, resulting in a high fluorescence polarization value. Test compound displacing the Fluormone GR Red from the complex causes a decrease in polarization. Mixture of GR recombinant protein, Fluormone GR Red, and a test compound was incubated at room temperature for 3 h 30 min and fluorescence polarization was measured using the FlexStation 3 Multi-Mode Microplate Reader (Molecular Devices). The shift in polarization values was used to determine the relative affinity of the test compound for the GR.

### Co-Immunoprecipitation of GR dimeric complex

HT-22 cells were co-transfected with GFP-tagged GR (Origene, # RG220189) and Myc-tagged GR (Origene, # RC220189) plasmid constructs using Lipofectamine 3000 reagent (Invitrogen) according to the manufacturers’ instruction. After 24 h, cell culture media was replaced with serum-free DMEM/High Glucose and then incubated at 37°C for 12 h for serum deprivation. Proteins were co-immunoprecipitated from whole-cell lysates of HT-22 cells, transfected with GFP-GR and Myc-GR, after stimulation with 10μM dexamethasone and 5μM PDE5 inhibitors (mirodenafil, sildenafil, tadalafil, vardenafil) for 6 h. Briefly, cells were harvested by scraping and cell pellets after centrifugation at 1000×*g* for 4 min were incubated in IP lysis buffer [20 mM Tris-Cl (pH 7.5), 150 mM NaCl, 1 mM EDTA, 1% Triton X-100, 10% glycerol, Complete Mini Protease Inhibitor cocktail, Pierce™ Phosphatase Inhibitor Mini Tablets] for 15 min on ice [25]. Cell lysates were centrifuged at 15,000×g at 4 °C for 10 min, and proteins in cleared lysates were co-immunoprecipitated by incubating at 4°C overnight with mouse anti-Myc antibody (9B11) (Cell Signaling Technology). Immunoprecipitates were incubated at 4°C for 1 h with Protein G Agarose (Roche) and washed with IP lysis buffer. Proteins in immune complexes were eluted in sodium dodecyl sulfate (SDS) sample buffer, fractionated by SDS-PAGE (polyacrylamide gel electrophoresis), transferred to PVDF membranes, and immunoblotted with the appropriate antibodies.

### Immunofluorescence confocal microscopy

HT-22 cells on poly-D-lysine coated coverslips were transfected with GFP-GR and Myc-GR using Lipofectamine 3000 reagent. After 24 h, culture media was exchanged with serum-free DMEM/High Glucose and then incubated at 37°C for 12 h for serum starvation. After treatment with 10μM dexamethasone and 5μM PDE5 inhibitors for 6 h, the cells were fixed in 4% paraformaldehyde in PBS for 10 min and then permeabilized with 0.1% Triton X-100 in PBS for 10 min at room temperature. The fixed HT-22 cells were blocked with 10% normal goat serum (Abcam) for 1 h at room temperature and incubated at 4 °C overnight with anti-Myc tag antibody (9B11) (Cell Signaling Technology). After washing with PBS, the cells were incubated with goat anti-mouse lgG H&L Alexa 647 (Abcam) at room temperature for 1 h and then washed with PBS. Coverslips were mounted with mounting medium with DAPI (Vector Laboratories). Fluorescence confocal images were obtained using LSM 800 confocal laser scanning microscopy (Zeiss). DAPI was used for nuclear staining. Mean fluorescence intensity in the nuclei and whole cells was measured using Zen Blue Edition software (Zeiss).

### Intracellular calcium measurement in primary human brain pericytes

Primary human brain pericytes were cultured in black 96-well plates according to the manufacturer’s instructions. After washing with HEPES-buffered saline (132 mM NaCl, 5.9 mM KCl, 1.2 mM MgCl_2_, 1.5 mM CaCl_2_, 11.5 mM Glucose, 11.5 mM HEPES, 1.2 mM NaH_2_PO_4_), 1 μM Fura-2 calcium indicator (Invitrogen) in HEPES-buffered saline was added to the pericytes in each well. After incubation with Fura-2 for 30 min, the pericytes were washed with HEPES and treated with H_2_O_2_ and AR1001 for 30 min. The intracellular calcium contents were measured using a 340/380 nm fluorescence excitation/emission ratio.

### Statistical analysis

Data are presented as the mean ± standard error of the mean (SEM). The n values in each figure correspond to the number of independent experiments or mice used. For qRT-PCR and other in vitro experiments, the data are expressed as relative fold-changes. Analysis of variance (ANOVA) with post hoc tests was used for multiple comparisons after confirming a normal distribution using the Shapiro-Wilk test. Significance was assessed with one-way ANOVA followed by Bonferroni’s post hoc comparison.

## Results

### Mirodenafil enhances the cognitive-behavioral performance in transgenic AD mice

**Fig. 1:**
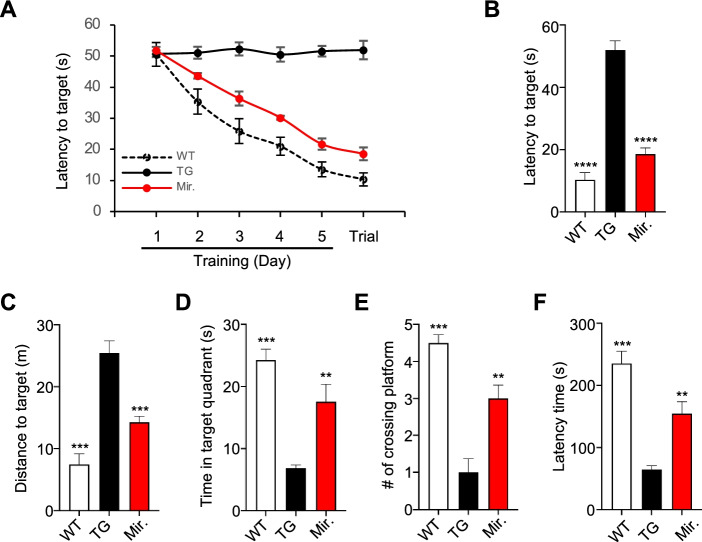
Mirodenafil improves cognitive function in the APP-C105 AD mice. **A** Mean latencies for the training session and visible platform trials in the Morris water maze (*n* = 6) **B**–**D** Morris water maze test was performed to test hippocampus-dependent learning and memory functions in 13-month-old male vehicle-treated wild-type (WT), vehicle-treated APP-C105 transgenic (TG), and mirodenafil-treated APP-C105 transgenic (Mirodenafil) mice, after 4 weeks of administration. Mirodenafil (4 mg/kg) in PBS was administered daily via intraperitoneal (i.p.) injection to 13-month-old male APP-C105 transgenic mice (*n* = 6) for 4 weeks. PBS vehicle solution was administered to age-matched male WT (*n* = 6) and transgenic (*n* = 6) mice. **B** Latency to target, **C** distance to reach target, **D** time in target quadrant, and **E** number of platform crossings were measured. To evaluate fear-conditioned learning and memory, APP-C105 mice were subjected to the passive avoidance test, and mirodenafil was administered by i.p. injection (4 mg/kg) for 4 weeks before the training trial. **F** Passive avoidance test. Latency to enter dark the compartment among WT, TG, and mirodenafil mice. Data are expressed as mean ± SEM; *n* = 4; **p < 0.01; ***p < 0.001; ****p < 0.0001. Statistical significance was assessed by one-way ANOVA followed by Bonferroni’s post hoc test for multiple comparisons

Mirodenafil was administered to APP-C105 mice daily for 4 weeks prior to the initiation of the 5-day Morris water maze training session. The mean latency to reach the target platform significantly decreased over the 5-day training period for wild-type and mirodenafil-treated APP-C105 transgenic mice, whereas vehicle-treated APP-C105 transgenic mice showed no changes in latency to reach the target over the course of the training session (Fig. 1A). Vehicle-treated APP-C105 transgenic mice exhibited severe memory impairment, as demonstrated by a significantly longer latency time, longer swimming distance prior to reaching the target, a shorter time spent in the target quadrant, and a smaller number of platform crossings. By contrast, the APP-C105 transgenic mice treated with mirodenafil displayed a significantly shorter swimming time by 64% decrease and swimming distance prior to reaching the target by 44% decrease, a longer time spent in the target quadrant by 256% increase, and a higher number of platform crossings compared with those for vehicle-treated APP-C105 mice by 300% increase (**p<0.01, ***p<0.001; Fig. 1B–E). In the passive avoidance test, vehicle-treated APP-C105 transgenic mice showed a significantly shorter latency to enter the dark compartment compared with vehicle-treated wild-type mice. By contrast, mirodenafil-treated APP-C105 transgenic mice exhibited a significant increase by 240% in the latency to enter the dark compartment compared with vehicle-treated APP-C105 transgenic mice (**p<0.01, ***p<0.001; Fig. 1F).

### Mirodenafil exerts neuroprotective functions via activating the cGMP/PKG/CREB signaling pathway

**Fig. 2:**
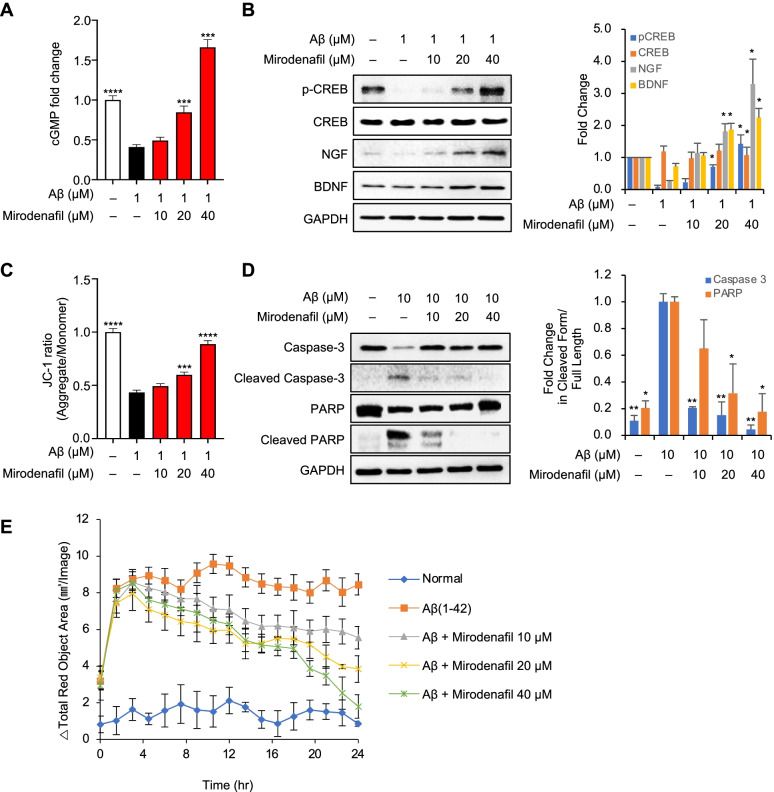
Mirodenafil exerts neuroprotective effects by activating the cGMP/PKG/CREB pathway. SH-SY5Y cells were treated with Aβ_42_ and increasing concentrations of mirodenafil. **A** Measurement of intracellular cGMP levels via ELISA. **B** Western blot analysis and semiquantitative measurements for total CREB, phosphorylated CREB (p-CREB), NGF, and BDNF. (*n* =3) **C** Assessment of mitochondrial membrane potential by tracking JC-1 dye. **D** Western blot analysis and semiquantitative measurements for the apoptotic markers caspase-3 and PARP. (*n*=3) **E** IncuCyte® live-cell imaging analysis to measure cell viability. Data are presented as the mean ± SEM; *n* ≥ 5; *P<0.05, **P < 0.01, ***P < 0.001. All statistical comparisons were performed relative to Aβ_42_-treated cells. Statistical significance was assessed by one-way ANOVA followed by Bonferroni’s post hoc test for multiple comparisons

Cells treated with oligomeric Aβ_42_ alone presented significantly reduced cGMP expression levels compared with untreated cells, whereas treatment with 20 μM mirodenafil significantly increased cGMP levels by about 200% in a dose-dependent manner (***p<0.001; Fig. 2A). The upregulation of cGMP-dependent PKG levels results in the increased activation of CREB by increasing phosphorylation at the regulatory site, serine 133 [26]. The western blot analysis of whole-cell lysates from SH-SY5Y cells treated with either Aβ_42_ alone or Aβ_42_ combined with mirodenafil showed that treatment with Aβ_42_ alone reduced CREB phosphorylated at serine 133, whereas the combined treatment with mirodenafil reversed the Aβ-induced decrease in phosphorylated CREB in a dose-dependent manner (Fig. 2B), consistent with the effects of mirodenafil on cGMP levels. The expression levels of nerve growth factor (NGF) and brain-derived neurotrophic factor (BDNF), which are both downstream effectors of CREB [27], were significantly increased following treatment with mirodenafil (Fig. 2B).

### Mirodenafil enhances neuronal survival by protecting the mitochondrial membrane potential and inhibiting apoptosis

Mirodenafil upregulated the levels of phosphorylated CREB; therefore, we examined whether mirodenafil also affected mitochondrial CREB activity. After treating SH-SY5Y cells with oligomeric Aβ_42_, either alone or in combination with mirodenafil, changes in the mitochondrial membrane potential were monitored through JC-1 staining. The combined treatment with mirodenafil restored the mitochondrial membrane potential to that of untreated cells (***p<0.001; Fig. 2C). Mitochondrial depolarization has been reported to induce cell death through the mitochondria-dependent intrinsic apoptotic pathway. After treating SH-SY5Y cells with Aβ_42_, either alone or in combination with mirodenafil, we observed the expression levels of apoptotic markers, including cleaved caspase-3 and cleaved poly ADP-ribose polymerase (PARP), through western blot analysis. As expected, treatment with Aβ_42_ alone increased the levels of cleaved caspase-3 and cleaved PARP, whereas the combined treatment with mirodenafil markedly reduced the expression levels of both apoptotic markers (Fig. 2D). The neuroprotective effects of mirodenafil were then examined by observing SH-SY5Y cell death in real-time, following treatment with Aβ_42_, either alone or in combination with mirodenafil, using IncuCyte Cytotox Red Reagent. The results showed that treatment with mirodenafil reduced Aβ_42_-induced apoptosis in SH-SY5Y cells in a dose-dependent manner (Fig. 2E).

### Mirodenafil reduces the Aβ and phosphorylated tau burdens in vivo

**Fig. 3:**
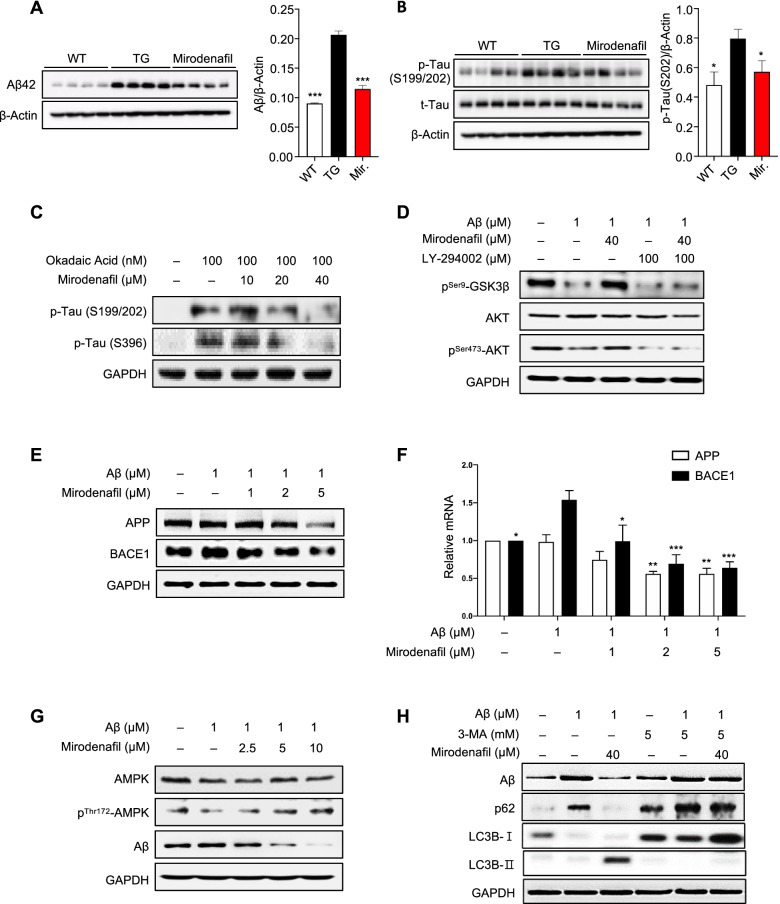
Mirodenafil downregulates GSK-3β signaling, resulting in reduced tau phosphorylation, decreased Aβ production by inhibiting amyloidogenesis and activating the autophagosomal pathway. **A**, **B** Western blot analysis for **A** Aβ, **B** phosphorylated tau, and total tau in mouse hippocampal homogenates of the APP-C105 mice (*n*=4). **C** Western blot analysis for phosphorylated tau at S199/202 and at S396 in SH-SY5Y cells after co-treatment with okadaic acid and mirodenafil (*n*=4). **D** Western blot analysis for phosphorylated GSK-3β and phosphorylated Akt in SH-SY5Y cells after co-treatment with Aβ_42_, mirodenafil, the PI3K inhibitor LY-294002 (*n*=4). **E** Western blot analysis and **F** real-time qRT-PCR results for APP and BACE1 expression after co-treatment with Aβ_42_ and mirodenafil in HT-22 cells (*n* ≥ 5). **G** Western blot analysis for phosphorylated AMPK and Aβ after co-treatment with Aβ_42_ and mirodenafil in SH-SY5Y cells (*n*=4). **H** Western blot analysis for the autophagy markers p62 and LC3B after co-treatment with Aβ_42_, mirodenafil, and autophagy inhibitor 3-MA (*n*=4). Data are presented as the mean ± SEM; *P < 0.05, **P < 0.01, ***P < 0.001, ****P < 0.0001. All statistical comparisons were performed relative to Aβ_42_-treated cells. Statistical significance was assessed by one-way ANOVA followed by Bonferroni’s post hoc test for multiple comparisons

The APP-C105 mice were treated with mirodenafil (4 mg/kg, *n* = 4), compared with vehicle-treated APP-C105 transgenic (*n* = 4) and wild-type mice (*n* = 4). The western blot analysis of the hippocampal tissue homogenates showed that the Aβ_42_ levels were significantly reduced by 45% in the hippocampi of mirodenafil-treated mice compared with those in the vehicle-treated mice (***p<0.001; Fig. 3A). In addition, the levels of tau phosphorylated at serines 199 and 202 were also decreased by 16% in the APP-C105 transgenic mouse hippocampus after administration with mirodenafil (*p<0.05; Fig. 3B).

### Mirodenafil inhibits GSK-3β signaling, resulting in reduced tau phosphorylation, impaired amyloidogenesis, and autophagosomal clearance of Aβ burden

In SH-SY5Y cells treated with the protein phosphatase inhibitor okadaic acid, treatment with mirodenafil reduced the phosphorylation of tau at serines 199 and 202, potentially phosphorylated by GSK-3β (Figs. 3C and S1A). This result was consistent with our previous observation that mirodenafil treatment reduced the level of tau phosphorylation at serines 199 and 202 in the hippocampus of APP-C105 mice (Fig. 3B).

To examine whether Akt signaling is involved in the phosphorylation of GSK-3β, SH-SY5Y cells were treated with oligomeric Aβ_42_, either alone or in combination with mirodenafil and GSK-3β phosphorylation was assessed by western blot analysis. Treatment with Aβ_42_ markedly decreased the levels of GSK-3β phosphorylation at serine 9 and diminished the levels of Akt phosphorylation at serine 473, whereas the combined treatment with mirodenafil fully restored phosphorylation of both GSK-3β and Akt (Figs. 3D and S1B) to that of untreated cells.

Alterations in APP and BACE1 expression levels were also tested in murine HT-22 hippocampal neuronal cells, co-treated with oligomeric mouse Aβ_42_ and mirodenafil. The levels of both APP and BACE1 protein expression were reduced by mirodenafil treatment in a dose-dependent manner (Figs. 3E and S1C). Quantitative reverse transcription-polymerase chain reaction (qRT-PCR) was conducted to analyze changes in mRNA levels of APP and BACE1 using RNA obtained from HT-22 cells, co-treated with Aβ_42_ and mirodenafil. The results showed that mirodenafil inhibited the Aβ-induced upregulation of BACE1 and dose-dependently reduced the transcriptional levels of both APP and BACE1 by about 50% (*p<0.05, *p<0.01, ***p<0.001; Fig. 3F).

To test whether the levels of Aβ and AMPK phosphorylation at threonine 172, which is a marker for autophagy initiation are affected by mirodenafil, SH-SY5Y cells treated with oligomeric Aβ_42_ either alone or in combination with mirodenafil and the degree of AMPK phosphorylation was determined by western blot analysis. Mirodenafil increased the levels of phosphorylated AMPK while concomitantly decreasing Aβ levels in a dose-dependent manner (Figs. 3G and S1D). In addition, changes in autophagy biomarker expression levels were observed in SH-SY5Y cells treated with Aβ_42_ and mirodenafil in the presence of the autophagy nucleation inhibitor 3-methyladenine (3-MA). Mirodenafil treatment enhanced autophagic clearance, as demonstrated by enhanced levels of microtubule-associated protein 1 light chain 3 (LC3B)-II, a marker of autophagosome formation, and reduced levels of p62, a marker of phagocytic degradation (Figs. 3H and S1E). In 3-MA and mirodenafil-treated cells, both autophagosome formation and phagocytic degradation were inhibited and Aβ reduction was not observed.

### Mirodenafil inhibits the transcriptional activity of the glucocorticoid receptor

**Fig. 4:**
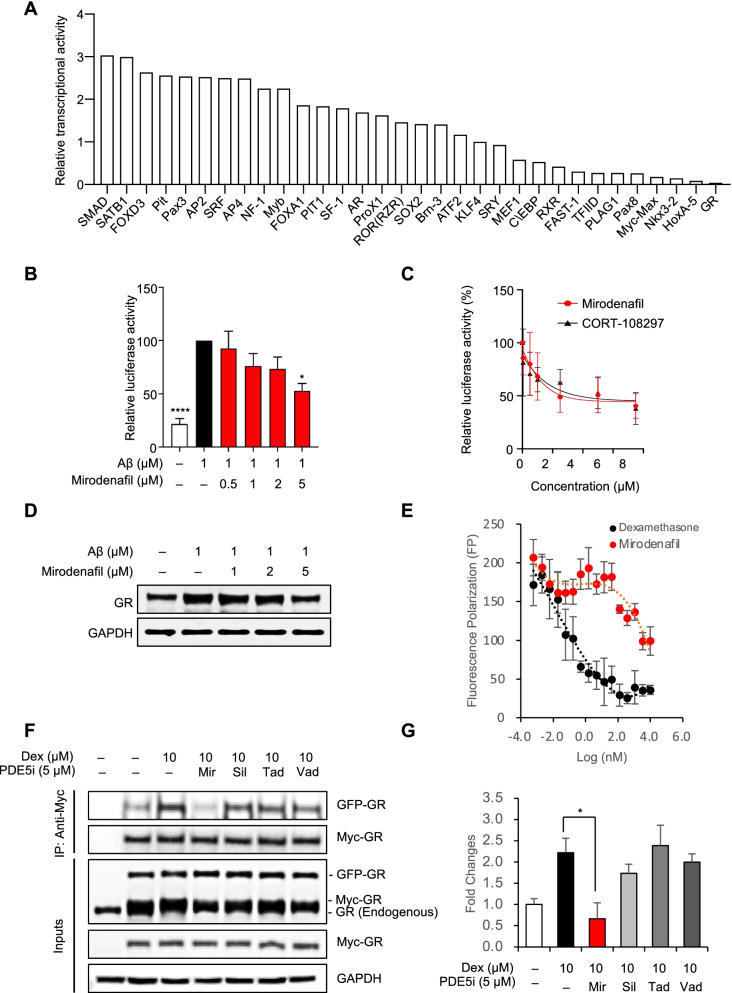
Mirodenafil inhibits transcriptional activity and homodimerization of GR. **A** TF profiling plate array was performed after treatment with Aβ and mirodenafil in HT-22 cells. **B** GR Dual-Luciferase reporter gene assay after treatment with Aβ and mirodenafil in HT-22 cells (*n* ≥ 5). **C** GR Dual-Luciferase reporter gene assay after co-treatment with dexamethasone and mirodenafil or CORT-108297 in HEK293 cells. **D** Western blot analysis for GR after co-treatment with Aβ_42_ and mirodenafil in HT-22 cells. **E** PolarScreen™ Glucocorticoid Receptor (GR) Competitor Assay to determine the IC_50_ values of dexamethasone and mirodenafil that directly bind to the full length of recombinant GR protein. **F** Co-immunoprecipitation of Myc-tagged GR with GFP-tagged GR in HT-22 cells after treatment with dexamethasone, mirodenafil (Mir), sildenafil (Sil), tadalafil (Tad), and vardenafil (Vad). **G** Quantifications of co-immunoprecipitation of GR homodimers after treatment with dexamethasone and PDE5 inhibitors using ImageJ software. Data are presented as the mean ± SEM; *P < 0.05, **P < 0.01, ****P < 0.0001. All statistical comparisons were performed relative to Aβ_42_- or dexamethasone-treated cells. Statistical significance was assessed by one-way ANOVA followed by Bonferroni’s post hoc test for multiple comparisons

To uncover further pivotal factors involved in the molecular networks underlying the polypharmacological effects of mirodenafil, a transcription factor (TF) activation profiling plate array was employed. Mirodenafil altered the transcription of multiple genes and significantly reduced the Aβ-mediated upregulation of glucocorticoid receptor (GR) transcription (Fig. 4A). Because the promoter region of the BACE1 gene overlaps with the DNA binding site of GR [28], and mirodenafil reduced expression levels of BACE1 (Figs. 3E and S1C), we tested whether mirodenafil downregulates BACE1 expression through the inhibition of GR transcriptional activity. GR transcriptional activity was significantly increased by oligomeric Aβ_42_ treatment alone and was suppressed upon co-treatment with mirodenafil in HT-22 cells (*p<0.05, ***p<0.001; Fig. 4B). In HEK293 cells responsive to dexamethasone-induced GR transcriptional activation, mirodenafil also inhibited GR transcriptional activity in a dose-dependent manner (Fig. 4C). Compared with a selective GR modulator, CORT-108297 (Fig. 4C), mirodenafil was determined to exert a similar level of GR antagonistic activity. Furthermore, GR expression levels were also decreased (Figs. 4D and S1F).

### Mirodenafil acts as a GR modulator, inhibiting homodimerization of GR

Due to the GR antagonistic activity of mirodenafil, competitive binding of mirodenafil to GR was examined. Intriguingly, mirodenafil directly binds to GR with a significantly lower binding affinity than dexamethasone, displacing only about 50% of fluorescently-labeled GR ligands (Fig. 4E). This atypical binding curve is reminiscent of the GR binding curve of Compound A (CpdA), which is a non-steroidal selective GR modulator (SEGRM). CpdA has been shown to prevent GR homodimerization, shifting GR transactivation toward transrepression [29]. Thus, we tested whether mirodenafil and other PDE5 inhibitors abrogate dexamethasone-induced GR homodimerization in HT-22 cells. Dexamethasone-induced formation of GR homodimeric complex was prevented by mirodenafil, and not by other PDE5 inhibitors (*p<0.05, Fig. 4F and G). Furthermore, formation of GR homodimers is required for their nuclear localization, leading to transcriptional activation via binding to the glucocorticoid response elements (GREs) [25]. As expected, treatment with dexamethasone fully facilitated translocation of GR dimers from the cytoplasm to nucleus, but mirodenafil partially prevented nuclear localization of GR dimers, consistent with the inhibitory effects of CpdA on nuclear localization of GR dimers [30] (Additional file 1: Fig. S2A and S2B)

### Mirodenafil activates the Wnt/β-catenin signaling pathway by downregulating Dkk1 expression

**Fig. 5:**
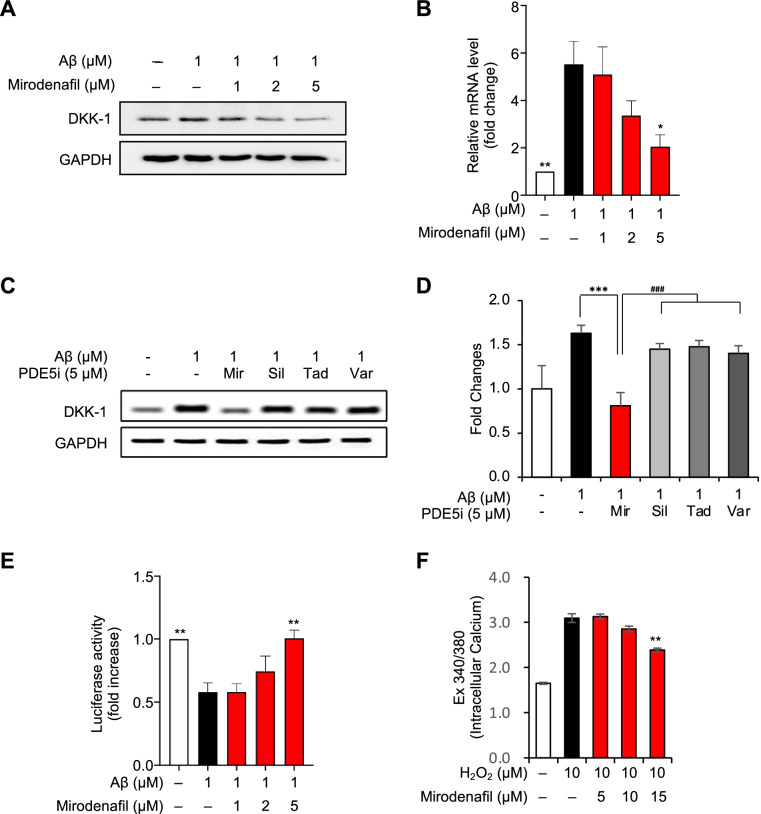
Mirodenafil reduces Dkk1 expression levels, activating the Wnt/β-catenin signaling. **A** Western blot analysis **B** Real-time qRT-PCR and for Dkk1 after co-treatment with Aβ_42_ and mirodenafil in HT-22 cells. **C** Comparative western blot analysis of Dkk1 expression levels after co-treatment with Aβ and PDE5 inhibitors [mirodenafil(Mir), sildenafil(Sil), tadalafil (Tad), vardenafil (Vad)]. **D** Quantification of Dkk1 western blots after co-treatment with Aβ and PDE5 inhibitors. **E** TOPFLASH reporter gene assay was employed to measure the levels of the Wnt/β-catenin transcriptional activity after co-treatment with Aβ_42_ and mirodenafil in HT-22 cells. **F** Measurement of intracellular Ca^2+^ contents in human brain pericytes after co-treatment with H_2_O_2_ and mirodenafil (*n* = 9). Data are presented as the mean ± SEM; *P < 0.05, **P < 0.01, ****P < 0.0001. All statistical comparisons were performed relative to Aβ_42_-treated cells. Statistical significance was assessed by one-way ANOVA followed by Bonferroni’s post hoc test for multiple comparisons

Previous reports have shown that Aβ increases the expression of Dickkopf-1 (Dkk1), a negative modulator of the Wnt/β-catenin pathway, resulting in synaptic loss and the upregulation of amyloidogenic APP processing [31]. Because Dkk1 is a primary target of GR transcriptional activity [32], we hypothesized that mirodenafil could inhibit the Aβ-mediated increase in Dkk1 expression by downregulating GR activity. Stimulation with Aβ_42_ alone significantly increased the transcription and expression of Dkk1, whereas co-treatment with mirodenafil markedly inhibited the Aβ-induced upregulation of Dkk1 expression (*p<0.05, **p<0.01; Fig. 5A, B). Unlike mirodenafil, other PDE5 inhibitors did not inhibit the homodimerization and nuclear localization of GR. Thus, to test whether sildenafil, tadalafil, and vardenafil reduce Dkk1 expression levels, increased by Aβ, Dkk1 expression levels were assessed by western blot analysis. As expected, other PDE5 inhibitors did not affect Dkk1 expression levels (***p<0.001; Fig. 5C, D). Dkk1 antagonizes the canonical Wnt/β-catenin pathway, which activates the expression of target genes in the nucleus by upregulating β-catenin/TCF-mediated transcription, and the inactivation of Dkk1 activates the canonical Wnt/β-catenin pathway [33]. Therefore, to examine whether mirodenafil downregulating Dkk1 expression could activate Wnt/β-catenin signaling, we performed a TOPLASH reporter assay after treatment with Aβ_42_, either alone or in combination with mirodenafil. We demonstrated that Aβ_42_ treatment inhibited β-catenin transcriptional activity, but the inhibitory effect of Aβ_42_ on β-catenin was significantly reversed by co-treatment with mirodenafil (**p<0.01; Fig. 5E). To test whether mirodenafil upregulates Wnt3a expression in HT-22 cells, qRT-PCR was employed. The mirodenafil-induced elevation of Wnt3a mRNA expression, a type-1 pro-canonical Wnt that selectively activates the Wnt/β-catenin signaling pathway, in Aβ_42_-treated HT-22 cells, but the expression of other Wnt ligands was not affected (*p<0.05; Fig. S3).

### Mirodenafil reduces intracellular Ca^2+^ levels in human brain pericytes

The Aβ-induced generation of reactive oxygen species reduces CBF due to the pericyte-mediated constriction of brain capillaries in a Ca^2+^-dependent manner [34]. Hydrogen peroxide increases intracellular Ca^2+^ levels, activating the contraction of brain pericytes [35]. Thus, the downregulation of hydrogen peroxide-induced intracellular Ca^2+^ spikes is likely to result in pericyte relaxation, increasing CBF. To test whether mirodenafil inhibits hydrogen peroxide-induced intracellular Ca^2+^ spikes, intracellular Ca^2+^ levels in human brain pericytes were monitored following co-treatment with hydrogen peroxide and mirodenafil. Mirodenafil significantly decreased the hydrogen peroxide-induced intracellular Ca^2+^ spike (**p<0.01; Fig. 5F).

## Discussion

Because AD is a multifactorial disorder that affects a broad network of signaling pathways, leading to cognitive deficits and multiple pathological mechanisms, effective treatment for AD will likely require the modulation of the multiple factors that are involved in the progression of AD. Here, we report that mirodenafil exerts neuroprotective effects against cognitive dysfunction and AD pathophysiology through multiple mechanisms of action involving (1) the reduction of Aβ and phosphorylated tau burdens, (2) enhanced neuronal survival through the activation of the cGMP/PKG/CREB pathway and its downstream effectors and (3) the inactivation of GR transcriptional activity and the activation of the Wnt/β-catenin signaling pathway (Additional file 1: Fig. S4).

Although cGMP-dependent CREB activation and memory improvement have been observed following treatment with most PDE5 inhibitors, only a few PDE5 inhibitors have demonstrated reductions in either the amyloid or tau burden, which represent the two neuropathological hallmarks of AD [36]. We found that mirodenafil administration could decrease the levels of both Aβ and phosphorylated tau in the hippocampus of APP-C105 transgenic mice (Fig. 3A, B). Mirodenafil administration reversed memory dysfunction in APP-C105 transgenic mice, as confirmed by the improved cognitive-behavioral performances during the Morris water maze and passive avoidance tests (Fig. 1A–F), suggesting that mirodenafil was able to rescue hippocampus-associated cognitive impairments in the AD model mice.

Our results suggested that the amyloid-targeting properties of mirodenafil modulate the production and clearance of Aβ. Mirodenafil could reduce amyloidogenesis by decreasing BACE1 and APP expression (Fig. 3E, F), indicating that the mirodenafil-induced downregulation of APP and BACE1 expression could contribute to the observed reduction in the Aβ burden observed in the brains of APP-C105 transgenic mice treated with mirodenafil. Furthermore, mirodenafil could promote Aβ clearance through autophagy-lysosome pathway activation, as indicated by the concomitant reduction in Aβ levels with the upregulation of autophagy-related proteins in SH-SY5Y cells (Fig. 3G–H and S1D–E). These results are interesting because it has been shown that inhibition of PDE5 by tadalafil or sildenafil stimulates cytosolic proteasomes, protein ubiquitination, and overall protein degradation without affecting autophagy in SH-SY5Y cells [37]. Thus, unlike other PDE5 inhibitors, mirodenafil may upregulate both the autophagy-lysosome pathway and the ubiquitin-proteasome system (UPS) for efficient degradation of toxic proteins, resulting in improved cognitive functions. Moreover, mirodenafil is about 10 times more potent than sildenafil in terms of IC_50_ values and is expected to have better efficacy on PDE5 inhibition than other PDE5 inhibitors, including sildenafil and tadalafil [38].

PDE5 inhibitors have previously been shown to activate the cGMP/PKG/CREB signaling pathway, resulting in enhanced neuronal cell survival and improved memory functions in transgenic AD mouse models [6, 9, 36]. In concurrence with previous reports, mirodenafil treatment reversed the Aβ_42_-induced downregulation of CREB and exerted neuroprotective effects by affecting the downstream targets of CREB, resulting in the upregulation of neurotrophic factors, the maintenance of the mitochondrial membrane potential, and the prevention of apoptotic cell death (Fig. 2). The neurotrophic factors affected by mirodenafil included BDNF, a neurotrophin known to be critical for neurogenesis and long-term memory, and NGF, a neuronal survival factor that promotes the transcription of anti-apoptotic factors [39]. Mirodenafil might also upregulate the levels of phosphorylated CREB in the mitochondrial matrix, promoting the transcription of the cyclic AMP response elements that are responsible for mitochondrial membrane integrity maintenance [40]. As a result, mirodenafil reduced the expression of apoptotic markers and inhibited Aβ_42_-mediated apoptosis in SH-SY5Y cells. These findings indicated that mirodenafil could activate the cGMP/PKG/CREB signaling pathway and increase the levels of downstream factors regulated by CREB transcriptional activity, impaired by Aβ_42_.

Using a TF activation profiling plate array to examine HT-22 cells treated with Aβ_42_ and mirodenafil, it was discovered that mirodenafil possesses antagonistic properties against GR (Fig. 4A–C), which led to the finding that mirodenafil could reverse the Aβ_42_-induced upregulation of Dkk1. In addition, mirodenafil prevented the formation of GR homodimers and antagonizes GR transcriptional activity, (Fig. 4B–C, F–G). GR transcriptional activity has been linked to the nuclear localization of GR dimers. CpdA, inhibiting GR dimerization, partly prevents dexamethasone-induced nuclear localization of GR dimers [30]. Consistent with this observation, mirodenafil partially inhibits the nuclear localization of GR dimers, unlike other PDE5 inhibitors. Dysregulation of GR signaling in the hypothalamic-pituitary-adrenal (HPA) axis plays central roles in the pathophysiology of AD. GR antagonists, which neutralize the HPA axis dysregulation, have been tested as a therapeutic agent for AD, but many GR antagonists have undesired side effects, limiting their therapeutic potential [41]. Thus, our results suggest that mirodenafil could be used as a polypharmacological GR modulator for AD, unlikely to cause unwanted adverse effects.

The canonical Wnt signaling antagonist Dkk1, which contain GREs in their promoter regions [32]. Accumulating evidence has indicated that inactivation of the Wnt/β-catenin signaling plays a pivotal role in Aβ-mediated neurodegeneration, and Dkk1 has become an attractive therapeutic target for AD [42]. Dkk1 expression was found to be increased in degenerating neurons obtained from post-mortem brain tissues of AD patients [43], and the restoration of Wnt/β-catenin signaling by reducing Dkk1 expression resulted in improved long-term memory and the reversal of synaptic degeneration [44]. Furthermore, the Aβ_42_-induced increase in Dkk1 expression has been shown to promote the amyloidogenic processing of APP by switching from canonical to noncanonical Wnt signaling, creating a positive feedback loop that increases Aβ production [31]. Therefore, the mirodenafil-induced downregulation of Dkk1 expression may suppress AD pathology by restoring Wnt/β-catenin signaling.

Brain capillaries form a microvascular network to exchange molecules between the blood and brain across the blood-brain barrier (BBB). Pericytes are perivascular cells that encapsulate brain capillaries and modulate capillary blood flow through Ca^2+^-dependent contractions [7]. Mirodenafil downregulated elevated levels of intracellular Ca^2+^ induced by hydrogen peroxide in human brain pericytes, consistent with a previous observation that NO/cGMP signaling activation inhibits reductions in intracellular Ca^2+^ levels, resulting in pericyte and brain capillary relaxation [34, 45] (Fig. 5F). This finding suggests that mirodenafil may increase CBF by relaxing pericytes in the brain capillary network.

Taken together, these results showed that mirodenafil has potential for use as a polypharmacological drug against AD pathology through the modulation of the cGMP/PKG/CREB pathway, Akt/GSK-3β-mediated phosphorylation of tau, GR transcriptional activity, Wnt/β-catenin signaling, and clearance of Aβ and tau. Although these targets have all been demonstrated to be effective against AD during preclinical stages, no disease-modifying orally administered drug has been approved for clinical AD treatment. Various clinical trials have resulted in discontinuation due to the lack of efficacy or severe side effects [46]. For example, several large-scale trials for BACE1 inhibitors were recently halted due to the worsening of cognition or toxicity [47]. Drawing on the recent failures faced during the clinical trials, we can deduce that a single target may be insufficient to stop the entirety of the complex pathophysiology associated with AD, and strong single factor-targeted drugs may induce severe side effects by disrupting homeostatic balance. Our study supports the use of a polypharmacological approach with repositioned drugs as an alternative to currently proposed therapies to successfully alleviate AD symptoms and pathology by acting on multiple mediating factors.

Despite the advantages proposed for the use of repositioned polypharmacological drugs, their clinical application must be approached with caution due to their low selectivity. One limitation of our study is directly associated with the polypharmacological activities of mirodenafil. As indicated by the TF profiling plate array results, mirodenafil affects numerous TFs, which we were unable to fully explore within the scope of this study. Further studies investigating the effects of these transcriptional alterations by mirodenafil and the resulting outcomes remain necessary.

## Conclusions

The findings presented here provide evidence showing that PDE5 inhibitor mirodenafil could be repositioned as a potential polypharmacological drug candidate to treat AD pathology. Mirodenafil displayed multiple mechanisms of action on multiple key molecular pathways involved in amyloid deposition, the cGMP/PKG/CREB pathway, GSK-3β kinase activity, GR transcriptional activity and the Wnt/catenin signaling. The therapeutic effects of mirodenafil were supported by the enhanced cognitive behavior and the reduction in both Aβ and phosphorylated tau levels in the mirodenafil-treated APP-C105 AD mouse model.

## Supplementary Information

**Additional file 1: Tables S1-S3.** **Figure S1.** Semiquantitative measurements for western blot analysis. (A) Tau phosphorylated at S199/202 and S396 in SH-SY5Y cells after co-treatment with okadaic acid and mirodenafil (n=4). (B) Phosphorylated GSK-3β and phosphorylated Akt in SH-SY5Y cells after co-treatment with Aβ_42_, mirodenafil, and the PI3K inhibitor LY-294002 (n=4). (C) APP and BACE1 expression after co-treatment with Aβ_42_ and mirodenafil in HT-22 cells (n ≥ 5). (D) Phosphorylated AMPK and Aβ after co-treatment with Aβ_42_ and mirodenafil in SH-SY5Y cells (n=4). (E) The autophagy markers p62 and LC3B after co-treatment with Aβ_42_, mirodenafil, and autophagy inhibitor 3-MA (n=4) (F) GR after co-treatment with Aβ_42_ and mirodenafil in HT-22 cells. Data are presented as the mean ± SEM; *P < 0.05, **P < 0.01. All statistical comparisons were performed relative to Aβ_42_-treated cells. Statistical significance was assessed by one-way ANOVA followed by Bonferroni’s post hoc test for multiple comparisons. **Figure S2.** **A**. Mirodenafil impedes nuclear localization of GR homodimers after treatment with dexamethasone. HEK293 cells were transfected with Myc-tagged GR and GFP-tagged GR constructs. Following incubation for 24 h, Myc-tagged GR and GFP-tagged GR were detected by immunofluorescence confocal microscopy after co-treatment with dexamethasone and PDE5 inhibitors for 6 h. **B**. Quantification of nuclear localization of Myc-tagged GR and GFP-tagged GR after co-treatment with dexamethasone and PDE5 inhibitors. Immunofluorescence confocal images were obtained using LSM 800 confocal laser scanning microscopy (Zeiss). Mean fluorescence intensity in the nuclei and whole cells was measured using Zen Blue Edition software (Zeiss). Nuclear GR intensity was divided by total GR intensity to indicate relative distribution of Myc-GR and GFP-GR in nuclei and cytoplasm of HT-22 cells. (n>12). Data are presented as the mean ± SEM; *P < 0.05. All statistical comparisons were performed relative to Aβ_42_-treated cells. Statistical significance was assessed by one-way ANOVA followed by Bonferroni’s post hoc test for multiple comparisons. **Figure S3.** Real-time qRT-PCR for Wnts (Wnt1, Wnt3a, Wnt5a, and Wnt7a) after treatment with Aβ_42_ and mirodenafil for 6 h in HT-22 cells (*n* ≥ 5). Data are presented as the mean ± SEM; *P < 0.05, **P < 0.01. All statistical comparisons were performed relative to Aβ_42_-treated cells. Statistical significance was assessed by one-way ANOVA followed by Bonferroni’s post hoc test for multiple comparisons. **Figure S4.** Mirodenafil ameliorates AD pathology through multiple mechanisms of action. Representative schematic illustration of the possible mechanisms through which mirodenafil improves cognition and ameliorates AD pathology. Mirodenafil enhances neuronal survival through the activation of the cGMP/PKG/CREB signaling pathway, which upregulates the expression of neurotrophic factors, promotes the maintenance of the mitochondrial membrane potential, and prevents apoptotic cell death. The downregulation of GSK-3β activity contributes to the reduction of phosphorylated tau, whereas the amyloid-targeting properties of mirodenafil decrease the Aβ burden by modulating the production, aggregation, and clearance of Aβ. Additionally, mirodenafil activates Wnt/β-catenin signaling by downregulating the expression of the Wnt antagonist Dkk1 or repressing GR transcriptional activity.

## Data Availability

The data analyzed during the current study are available from the corresponding authors on reasonable request.
