# Supplementary material for: Phosphodiesterase 5 inhibitor mirodenafil ameliorates Alzheimer-like pathology and symptoms by multimodal actions
Source: Alzheimers Res Ther. 2022 Jul 8;14:92. doi: 10.1186/s13195-022-01034-3 (PMC9264543; doi:10.1186/s13195-022-01034-3)
Supplement: Supplementary file 1 — Additional file 1: Tables S1-S3. Figure S1. Semiquantitative measurements for western blot analysis. (A) Tau phosphorylated at S199/202 and S396 in SH-SY5Y cells after co-treatment with okadaic acid and mirodenafil (n=4). (B) Phosphorylated GSK-3β and phosphorylated Akt in SH-SY5Y cells after co-treatment with Aβ42, mirodenafil, and the PI3K inhibitor LY-294002 (n=4). (C) APP and BACE1 expression after co-treatment with Aβ42 and mirodenafil in HT-22 cells (n ≥ 5). (D) Phosphorylated AMPK and Aβ after co-treatment with Aβ42 and mirodenafil in SH-SY5Y cells (n=4). (E) The autophagy markers p62 and LC3B after co-treatment with Aβ42, mirodenafil, and autophagy inhibitor 3-MA (n=4) (F) GR after co-treatment with Aβ42 and mirodenafil in HT-22 cells. Data are presented as the mean ± SEM; *P < 0.05, **P < 0.01. All statistical comparisons were performed relative to Aβ42-treated cells. Statistical significance was assessed by one-way ANOVA followed by Bonferroni’s post hoc test for multiple comparisons. Figure S2. A. Mirodenafil impedes nuclear localization of GR homodimers after treatment with dexamethasone. HEK293 cells were transfected with Myc-tagged GR and GFP-tagged GR constructs. Following incubation for 24 h, Myc-tagged GR and GFP-tagged GR were detected by immunofluorescence confocal microscopy after co-treatment with dexamethasone and PDE5 inhibitors for 6 h. B. Quantification of nuclear localization of Myc-tagged GR and GFP-tagged GR after co-treatment with dexamethasone and PDE5 inhibitors. Immunofluorescence confocal images were obtained using LSM 800 confocal laser scanning microscopy (Zeiss). Mean fluorescence intensity in the nuclei and whole cells was measured using Zen Blue Edition software (Zeiss). Nuclear GR intensity was divided by total GR intensity to indicate relative distribution of Myc-GR and GFP-GR in nuclei and cytoplasm of HT-22 cells. (n>12). Data are presented as the mean ± SEM; *P < 0.05. All statistical comparisons were performed relative to [file 13195_2022_1034_MOESM1_ESM.pdf]

Figure S1

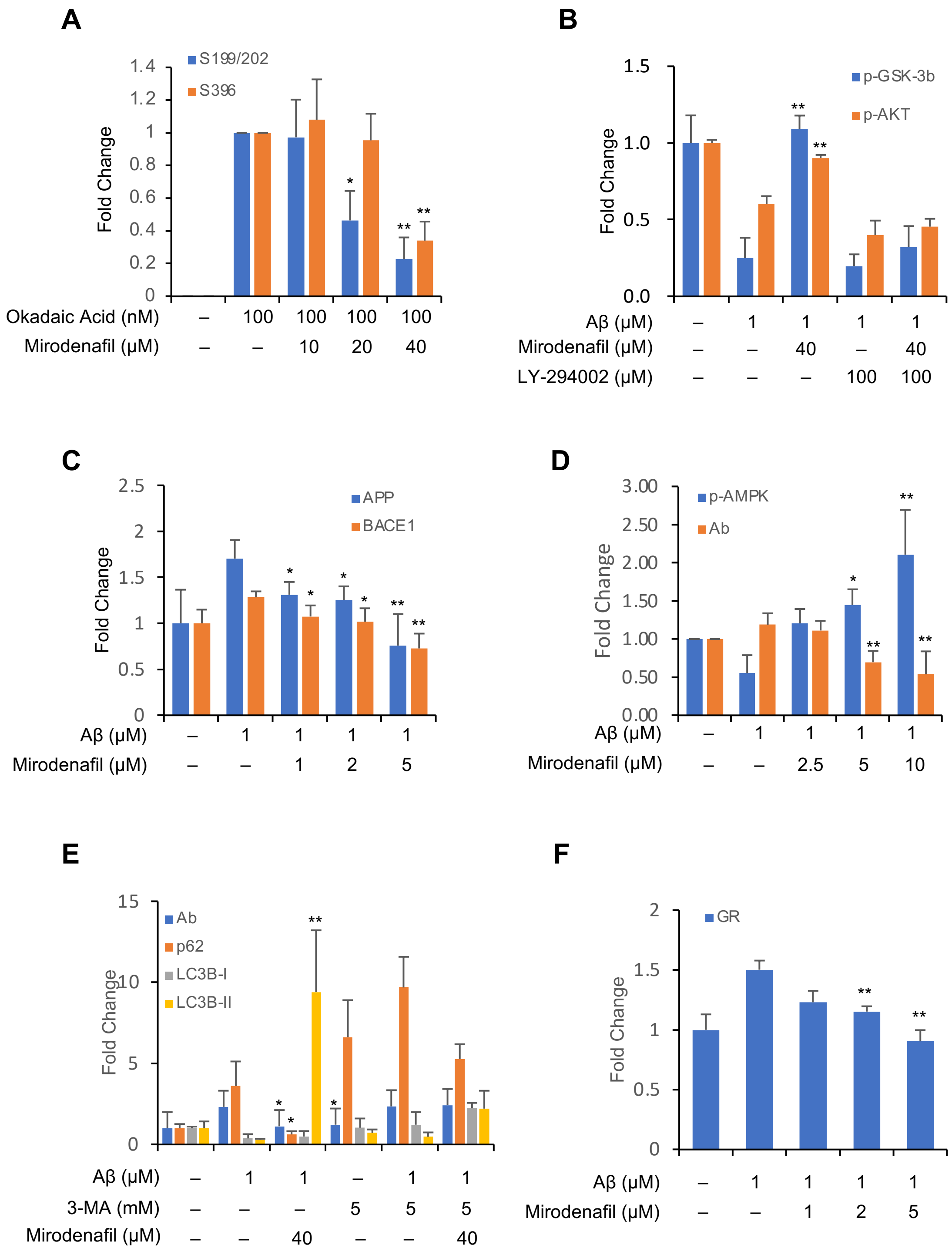
A
S199/202
S396
Fold Change
1.4
1.2
1
0.8
0.6
0.6
0.4
0.2
0
Okadaic Acid (nM) – 100 100 100 100
Mirodenafil (μM) – – 10 20 40
B
p-GSK-3b
p-AKT
Fold Change
1.5
1.0
0.5
0.0
Aβ (μM) – 1 1 1 1
Mirodenafil (μM) – – 40 – 40
LY-294002 (μM) – – – 100 100
C
APP
BACE1
Fold Change
2.5
2
1.5
1
0.5
0
Aβ (μM) – 1 1 1 1
Mirodenafil (μM) – – 1 2 5
D
p-AMPK
Ab
Fold Change
3.00
2.50
2.00
1.50
1.00
0.50
0.00
Aβ (μM) – 1 1 1 1
Mirodenafil (μM) – – 2.5 5 10
E
Ab
p62
LC3B-I
LC3B-II
Fold Change
15
10
5
0
Aβ (μM) – 1 1 – 1 1
3-MA (mM) – – – 5 5 5
Mirodenafil (μM) – – 40 – – 40
F
GR
Fold Change
2
1.5
1
0.5
0
Aβ (μM) – 1 1 1 1
Mirodenafil (μM) – – 1 2 5

Figure S2. A

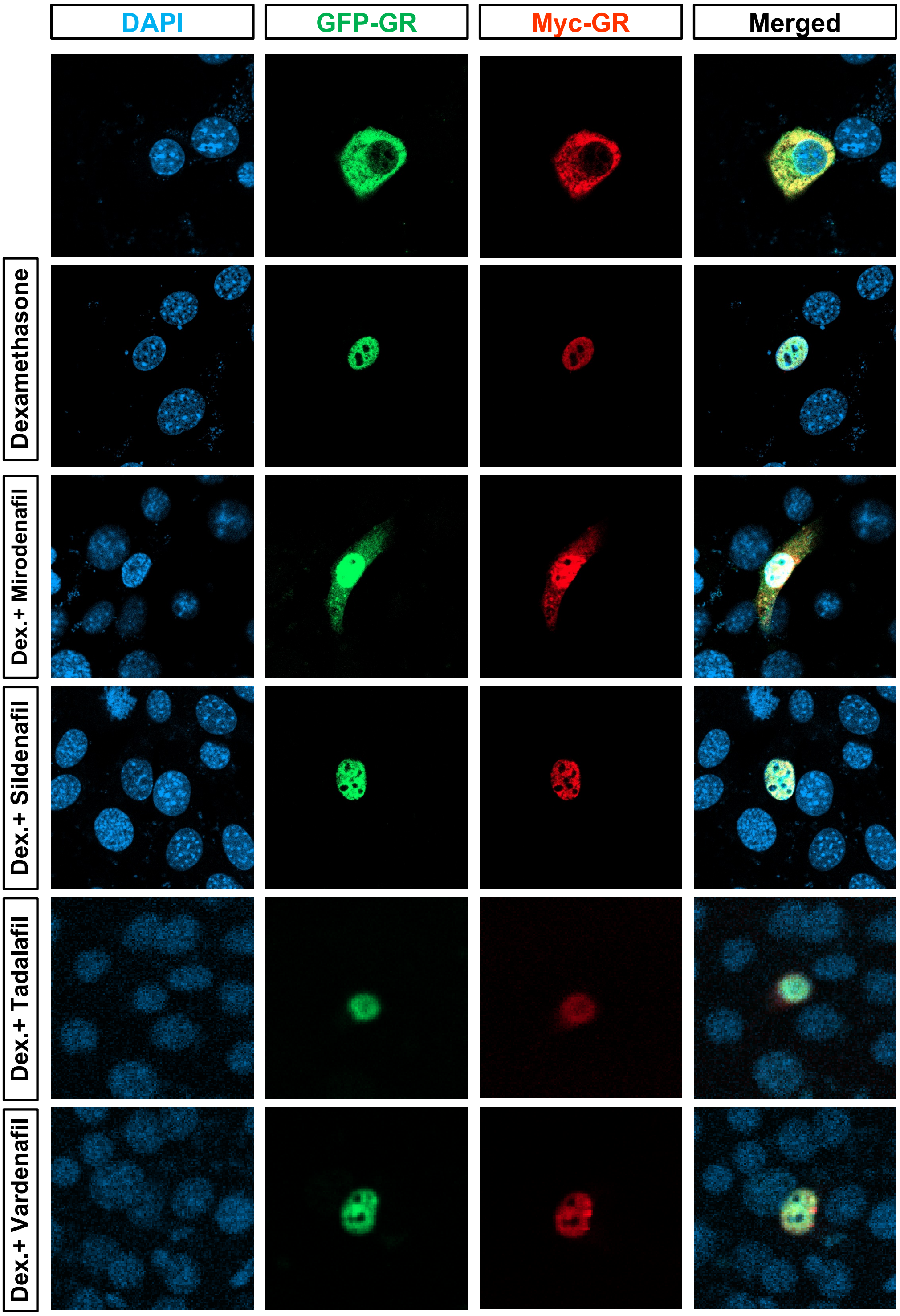

DAPI
GFP-GR
Myc-GR
Merged
Dexamethasone
Dex.+ Mirodenafil
Dex.+ Sildenafil
Dex.+ Tadalafil
Dex.+ Vardenafil

Figure S2. B

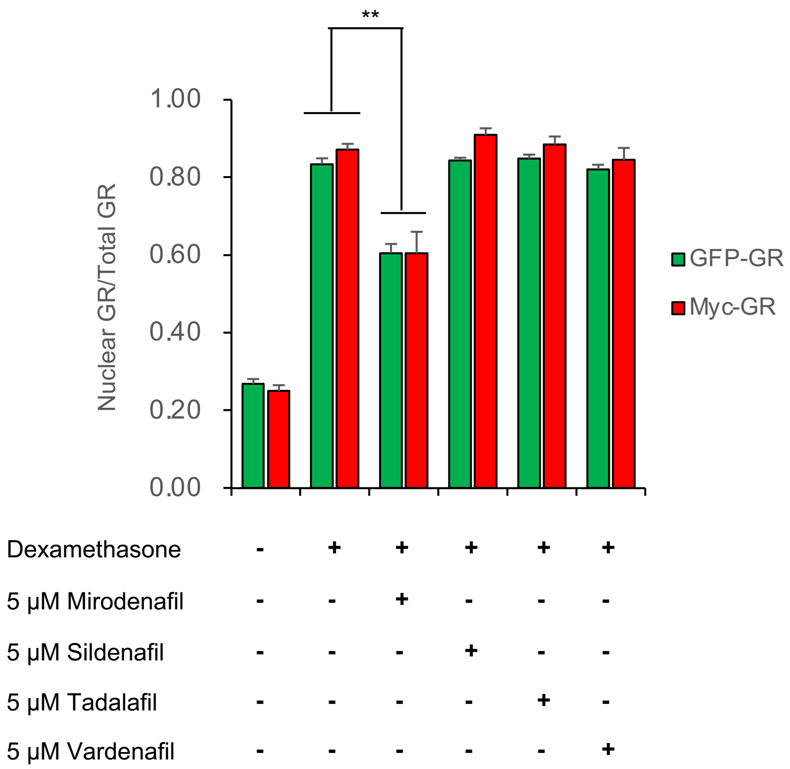

Figure S3

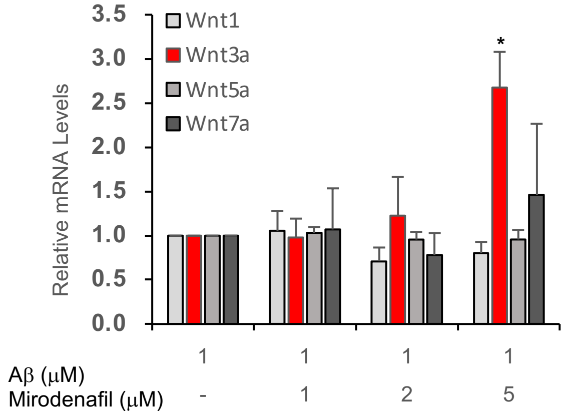

Figure S4

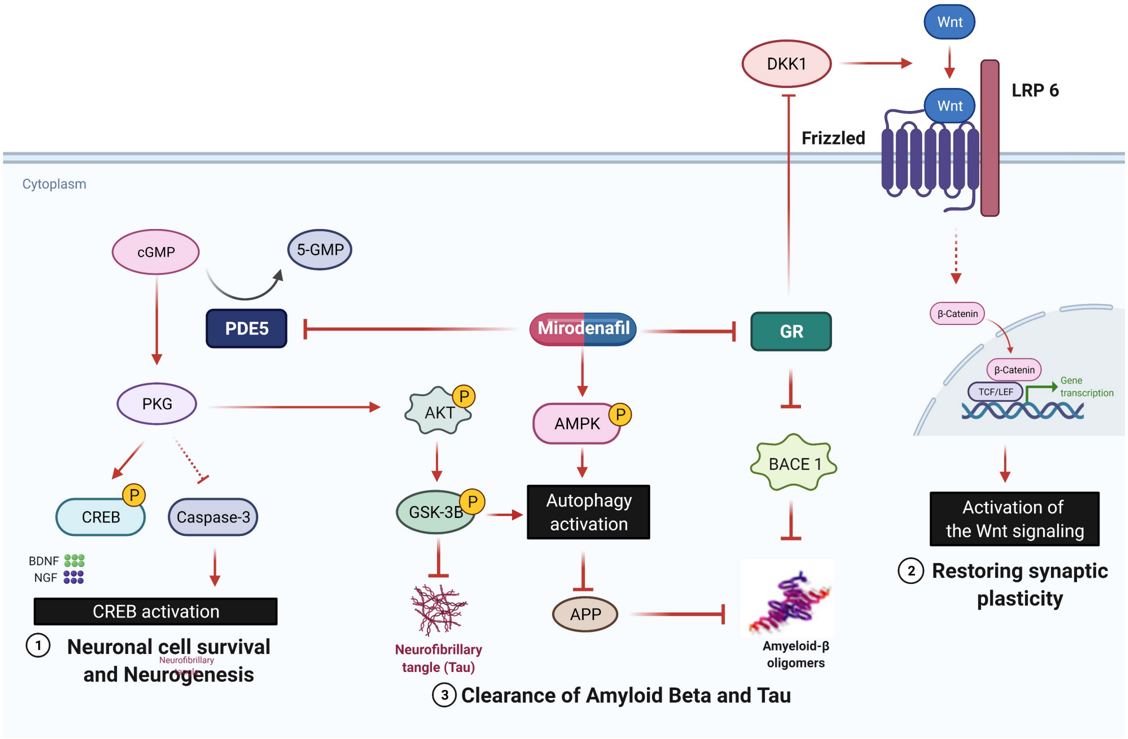

Wnt
DKK1
LRP 6
Wnt
Frizzled
Cytoplasm
cGMP
5-GMP
PDE5
Mirodenafil
GR
β-Catenin
β-Catenin
TCF/LEF
Gene transcription
PKG
AKT
P
AMPK
P
BACE 1
CREB
P
Caspase-3
GSK-3B
P
Autophagy activation
Activation of the Wnt signaling
BDNF
NGF
CREB activation
APP
2 Restoring synaptic plasticity
1 Neuronal cell survival and Neurogenesis
Neurofibrillary tangle (Tau)
Ameyloid-β oligomers
3 Clearance of Amyloid Beta and Tau

Table S1. Summary of general pharmacology study on the CNS in healthy mice

CNS Effects	Testing Method	Results compared with Vehicle Control Group
Spontaneous Locomotor Activity	Activity Monitor	No difference
Motor Coordination	Rotarod Test	No difference
Analgesia	Acetic Acid	No difference
	Hot Plate	No difference
Body Temperature	Rectal Temperature	No difference
Sleeping Time	Pentobarbital Sodium	No difference

Table S2. Primary antibodies used

Antibodies	Suppliers	Catalog No.	Species
Aβ 1-42	Abcam	ab12267	Rabbit
AKT	Cell Signaling Technology	9272	Rabbit
Phospho-Akt (Ser473)	Cell Signaling Technology	4060	Rabbit
AMPK	Cell Signaling Technology	2532	Rabbit
p-AMPK	Cell Signaling Technology	2531	Rabbit
APP	Abcam	ab32136	Rabbit
ATG5/ATG12	Abcam	ab109490	Rabbit
ATG7	Abcam	ab133528	Rabbit
BACE1	Cell Signaling Technology	5606	Rabbit
BDNF	Abcam	ab108319	Rabbit
β-ACTIN	Santa Cruz	sc-47778	Mouse
Caspase-3	Cell Signaling Technology	9662	Rabbit
Cleaved Caspase-3	Cell Signaling Technology	9661	Rabbit
p-CREB (phospho S133)	Abcam	ab32096	Rabbit
Dkk1	Abcam	ab61275	Mouse
GAPDH	Cell Signaling Technology	2118	Rabbit
GFAP	Millipore	AB5541	Chicken
Glucocorticoid Receptor	Cell Signaling Technology	3660	Rabbit
Phospho-GSK-3-beta (Ser9)	Cell Signaling Technology	9322	Rabbit
LC3B	Cell Signaling Technology	2775	Rabbit
NGF	Abcam	ab52918	Rabbit
p62	Cell Signaling Technology	39749	Rabbit
PARP	Cell Signaling Technology	9532	Rabbit
Tau	Cell Signaling Technology	46687	Rabbit
pSer199/202-Tau	Millipore	AB9674	Rabbit
pSer396-Tau	Abcam	ab109390	Rabbit

Table S3. PCR primers

Targets	Strand	Primer sequences	Size (bp)	T_m (°C)	Origin
APP	S	5'- CTACGGAAACGACGCTCTCATG -3'	209	59	Mouse
	AS	5'- CAGAACCTGGTCGAGTGGTCAG -3'			
BACE1	S	5'- GGAACCCATCTCGGCATCCGG -3'	215	59	Mouse
	AS	5'- ACCAGGATGTTGAGCGTCTGTG -3'			
β-ACTIN	S	5’-AGAAGCTGTGCTATGTTGCTCT -3’	191	53	Mouse
	AS	5’- AGTTTCATGGATGCCACAGGAT -3’			
DKK1	S	5'- TCTGCTAGGAGCCAGTGCC -3'	191	50	Mouse
	AS	5'- GATGGTGATCTTTCTGTATCC -3'			
WNT1	S	5'- CTCTTTGGCCGAGAGTTCGTGG -3'	251	60	Mouse
	AS	5'- CCTCGGTTGCCGTAAAGGACGC -3'			
WNT3A	S	5'- CTCGCATGGCATAGATGGGTGC -3'	153	60	Mouse
	AS	5'- GCAGGTGTGCACGTCATAGAC -3'			
WNT5A	S	5’-CATGGAGTGTCTGGCTCCTG -3'	279	60	Mouse
	AS	5'- GTCCATCCCCTCTGAGGTCTTG -3'			
WNT7A	S	5’- CGGGAGATCAAGCAGAATGC -3’	167	57	Mouse
	AS	5’- GCCTAGCTCTCGGAACTGTGGC -3’			
